# Effect and immediate after-effect of lightly gripping the cane on postural sway

**DOI:** 10.1186/s40101-016-0096-4

**Published:** 2016-05-18

**Authors:** Kazushige Oshita, Sumio Yano

**Affiliations:** 1 Department of Sports Science, Kyushu Kyoritsu University, 1-8 Jiyugaoka, Yahatanishi, Kitakyushu, 807-8585 Japan; 2 Graduate School of Human Development and Environment, Kobe University, 3-11 Tsu-rukabuto, Nada, Kobe, 657-8501 Japan

**Keywords:** Haptic input, Light touch phenomenon, Center of foot pressure, Co-contraction, Feedback, Motor learning

## Abstract

**Background:**

This study investigated the effect and after-effect of lightly touching a real cane on postural sway and ankle muscle activity.

**Method:**

Participants performed a single-leg stance (SLS) task with their eyes closed for 30 s under three tasks. In the first and third tasks, the participants performed a normal SLS. In the second task, the participants in light-grip group (*n* = 11) were asked to perform SLS while lightly gripping a cane with their hand. The participants in depend-on-cane group (*n* = 11) were asked to support their own body with a cane.

**Results:**

Postural sway during a single-leg stance is decreased by light gripping and is accompanied by decreased co-contraction of the ankle-joint muscles. If a participant lightly gripped a cane, postural sway decreased not only during the light gripping but also immediately after the withdrawal of the cane. Although postural sway and co-contraction in the depend-on-cane group were significantly decreased during the second task compared to the first task, they were not significantly changed between the first and third tasks.

**Conclusion:**

These results suggest that lightly gripped cane provides a haptic sensory cue that can be used to assist postural control mechanisms due to enhanced perception of self-motion through sensory interaction with the environment through the cane. Further, the haptic sensory cue during postural maintenance might be promoted as a practice effect of postural control.

## Background

Independent mobility is an important factor that influences quality of life, and better balance control is required to decrease the incidence of falls. Therefore, many researchers have attempted to increase balance control using various tools to prevent falls. One such tool, the cane, can easily support a person’s body weight. This can certainly help individuals ambulate independently while greatly reducing their risk of falling with progressive weakness of physical fitness (i.e., leg strength). However, cane use may weaken the physical fitness level of individuals who otherwise have adequate strength to maintain their own postures because it greatly reduces the muscular force outputs that are normally used to support body weight [1].

Researchers have found that providing additional haptic sensory input through the hand or finger decreases postural sway during quiet standing [2–6]. Jeka and Lackner [2] showed that lightly touching the tip of the index finger on a fixed surface at waist height (contact force levels that are insufficient for providing mechanical body support; <1 N) resulted in decreased postural sway during a quiet stance. These effects have also been observed for light touching of an unstable object; reportedly, lightly touching a mobile stick [7] or the upper part of one’s own thighs [8, 9] significantly decreases postural sway during a quiet stance. These findings indicate that lightly touching an object during quiet standing primarily provides information about the relative movement of the body segments and helps an individual sense the movements of the trunk, arms, and thighs relative to one another.

Furthermore, these light touch effects persist immediately after the withdrawal of light touching. Johannsen et al. [10] reported that decreases in postural sway during 5 s of light touch persisted even immediately thereafter. Oshita and Yano [9] also reported that postural sway was significantly decreased during 30 s of lightly touching one’s own thighs and that it tended to decrease thereafter. Therefore, these studies suggest that the haptic sensory cue during postural maintenance might be promoted as a practice effect of postural control. However, these previous studies did not examine the after-touch effect under various body support conditions. Because the after-touch effect observed only in light touching has not been examined, studies are needed to compare the after-effect of light touch (i.e., haptic sensory input) with that of heavy touch (i.e., mechanical body support).

Although strongly gripping and depending on a cane greatly reduces muscular force outputs to support one’s body weight [1], lightly touching or gripping a cane does not reduce muscular force outputs because contact force levels are insufficient for providing mechanical body support. Observation of the after-effects of lightly touching a cane would aid in the development of a useful application to acquire balance control ability. Further, the mechanisms underlying the association between postural sway and light touch have not been thoroughly examined. Therefore, studies are needed to clarify the direct relationship between muscle activity and the effect of light touch on balance control. Thus, the purpose of the current study was to investigate the effect and after-effect of lightly touching a real cane on postural sway and leg muscle activity.

## Methods

### Participants

Data were obtained from 22 healthy men (age, 19–26 years old; height, 1.63–1.83 m; weight, 55.3–72.6 kg) with no current or previous medical history of neural, muscular, or skeletal disorders. The participants were randomly assigned to light-grip (LG; *n* = 11) or depend-on-cane (DC; *n* = 11) group. Before participating, all participants provided informed consent after being explained the study purpose. This study was approved by the human ethics committee of the Graduate School of Human Development and Environment, Kobe University (project registration number 165).

### Materials and procedure

In this study, postural sway and muscular activity were evaluated during the single-leg stance (SLS) on each participant’s preferred leg with eyes closed since the participants were speculated to have the ability to remain still during a bi-pedal stance. Each participant identified the preferred leg as that he believed was stronger and with which he would kick a ball [11, 12]. This was the right side in all participants. If an individual kicks a ball with their preferred limb, the other limb is often required to support the entire body weight. Therefore, balance control ability might be different between limbs: the limb preferred for daily use or the other limb regularly supporting the body weight over many years. However, Hoffman et al. [13] reported no difference in unilateral postural sway (evaluated by total sway area and sway path length of the center of foot pressure) between the functionally preferred and non-preferred lower limbs in a healthy population of young adults.

During SLS, postural sway was evaluated by center of foot pressure (COP) using a force platform (T.K.K. 5810; Takei Scientific Instruments Co. Ltd., Japan). The force platform was connected to a personal computer by an analog-to-digital converter (AI-1608AY-USB; CONTEC, Japan). Data were recorded at 100 Hz and stored on a hard disk for later analyses.

To assess muscle activity during SLS, surface electromyography (EMG) data were collected from the gastrocnemius (GAS) lateralis and tibialis anterior (TA) muscles. These muscles were selected based on the study result that assessed muscle activity during a quiet stance [14]; although co-contraction of the lower-leg (ankle-joint) muscles was significantly increased with eyes closed during the SLS compared to the bi-pedal stance, no significant effect on the upper-leg (hip joint) muscles was seen in normal healthy participants. On the preferred leg, bipolar surface electrodes with an EMG amplifier (ID2PAD; Oisaka Electronic Equipment Ltd., Japan) were placed over the GAS and TA at a 2-cm inter-electrode distance. Skin impedance to the electrical signal was decreased by gentle abrasion with a skin preparation gel (Skin Pure; NIHON KODEN, Japan) and wiping with isopropyl alcohol swabs. The signals were amplified 500 times and acquired at a sampling frequency of 1000 Hz using a data logger with an analog-to-digital converter (LP-MS1002; Logical Product Corporation, Japan).

### Experimental protocol

Maximum voluntary muscle contractions (MVCs) were initially performed in the directions of ankle plantar flexion and dorsiflexion. To engage the GAS, each participant stood on a squat rack on his preferred leg while holding onto the rack to maintain his balance. The participants were instructed to attempt to rise up onto their toe against the pressure applied to their shoulders by the investigator. To engage the TA, each participant sat upright with his knees in full extension and 90° of ankle dorsiflexion was prevented. The participant was then instructed to attempt dorsiflexion against the applied pressure in the direction of ankle plantar flexion by the investigator. During the MVC tests, visual feedback of the EMG signals was displayed on a PC monitor and verbal encouragement was provided by the investigators. The participants were asked to perform each MVC twice for 5 s with a 10-s pause between tests. The participants were allowed to reject an effort that they deemed as not “maximal.”

**Fig. 1:**
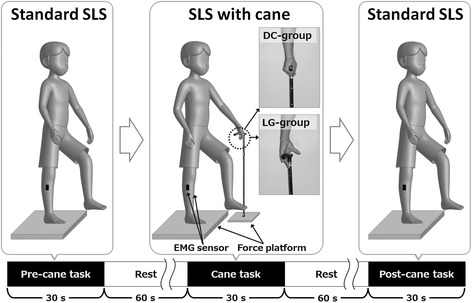
Schematic of the experimental protocol

After the MVC tests, the participants performed three SLS tasks with a 1-min rest period (Fig. 1). The duration of this rest period was based on a study that investigated the effects of light touch of the upper legs on postural sway [9]. The first and third tasks were standard SLS (pre- and post-cane tasks). All participants were instructed to remove all footwear, step onto a force platform, and maintain an upright stance. The experimenter then instructed the participant to close his eyes, raise his non-preferred leg (foot) from the platform, and maintain the posture for 30 s. This duration was based on a study that evaluated the muscular activity during the SLS [14]. Before the MVC tests, a practice session allowed the participants to become familiar with the SLS protocol for approximately 5 min.

In the second task (cane task), the participants in the LG group were asked to perform SLS and let their left hand lightly grip the cane (using fingers and palm as shown in Fig. 1 with no vertical force) in the left anterior oblique position for 30 s. During this task, the vertical force on the cane was measured by a platform scale with a digital indicator (HT-500, A and D Co., Ltd., Japan), and carefully observed by the investigator. If the vertical force exceed 1 N (equivalent to 0.14–0.22 % of body weight), the experiment trials (including the pre-cane task) were terminated and repeated after 30 min. The participants in the DC group were instructed to perform SLS while supporting their weight with the cane held in their left hand. The cane length was regulated so the top of the cane would reach the crease of the wrist while the participant stood up straight with his arms at his sides. The length was confirmed when each participant held the cane while standing with the elbow flexed 15–20°.

### Data analysis

To assess the COP from the stored force data, a 10-s period in the middle portion of each task was selected for the analysis since body motion was not immediately stabilized when the participants performed the SLS. The mean velocity of COP trajectory (V-COP) was calculated as the total path length of COP displacement (in mm) divided by calculated time (in s).

The stored EMG data were processed using the waveform analysis software SPCANA (ver. 4.92). After band-pass filtering (1–500 Hz), the root mean square of the EMG signal (RMS-EMG) in each task was calculated. In each MVC task, RMS was calculated every 0.2 s (200 samples), and the maximum 0.2-s interval RMS-EMG value from the two MVCs was recruited as the MVC value. In each SLS task, a 10-s period in the middle portion was selected to calculate the RMS-EMG value. Further, these data were normalized to the MVC value (% MVC) for the muscle activity evaluation. The co-contraction index (CCI) was calculated using the following equations [15] to evaluate co-contraction of the antagonist muscle. This index was recruited as reported in a study that evaluated the muscle activity during bi-pedal stance and SLS [14] as:

$$ \mathrm{C}\mathrm{C}\mathrm{I}=\left(\frac{\%\;{\mathrm{MVC}}_{\mathrm{lower}}}{\%\;{\mathrm{MVC}}_{\mathrm{higher}}}\right)\kern0.5em \times \left(\%\;{\mathrm{MVC}}_{\mathrm{lower}}+\%\;{\mathrm{MVC}}_{\mathrm{higher}}\right) $$

where % MVC lower and % MVC higher represent the average normalized RMS-EMG value of TA or GAS activities.

### Statistical analysis

V-COP, % MVC, and CCI between the three SLS tasks in each group were evaluated using two-way repeated-measures analysis of variance (ANOVA), which was used to compare the three tasks and the two groups. Relative changes in V-COP and CCI in the cane and post-cane tasks compared to the pre-cane task were also evaluated using two-way ANOVA, which was used to compare the tasks and the groups. After the ANOVA, post hoc multiple comparisons were made using Fisher’s least significant difference (LSD) test. To investigate the effect of the cane use condition on postural sway and muscle activity, the relationship between V-COP and CCI during the cane task was evaluated by regression analysis. Values of *P* < 0.05 were considered statistically significant. These analyses were performed using J-STAT (ver. 12.5) and js-STAR (ver. 2.0.6j) software. In addition to the significance testing, effect sizes (*r*) were calculated for changing tasks. Data are presented as mean ± standard deviation (SEM) unless otherwise stated.

## Results

**Fig. 2:**
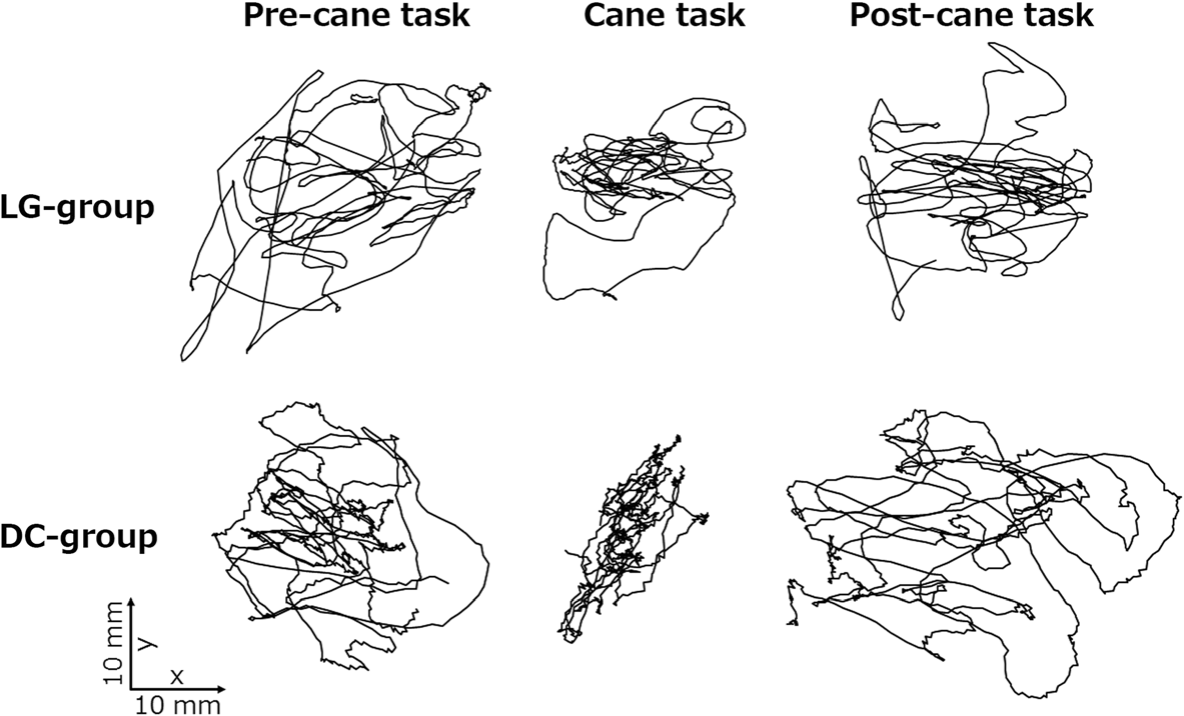
Individual trajectories of the center of foot pressure path in a representative sample from the light-grip (LG) and depend-on-cane (DC) groups

Figure 2 shows the representative trajectories of the COP path in each group. For the average V-COP in each group (Table 1), the factors of tasks and the interaction between tasks and groups exhibited statistically significant effects. Further, post hoc test revealed that postural sway in the LG group significantly decreased not only during the light gripping (second task) but also immediately after the withdrawal of the cane (third task). Although postural sway in the DC group was significantly decreased during the cane task compared to pre-cane task, immediate after-effect of withdrawal of the cane on postural sway was not observed. Effect sizes of V-COP were large in the cane and post-cane tasks versus the pre-cane task (*r* = 0.79 and 0.67) in the LG group. Although a large effect was seen in the cane versus pre-cane tasks (*r* = 0.82), a small effect in the pre- versus post-cane tasks (*r* = 0.07) was seen in the DC group. Therefore, postural sway is decreased by both, supporting a person’s own weight with a cane and just lightly gripping the cane. Further, if a participant lightly gripped a cane during SLS, postural sway decreased not only during the light gripping but also immediately thereafter. However, no after-effect was seen when the participant used the cane to support his weight.

**Table 1:** Mean value of each variable during three single-leg stance tasks

Variables	Group	Pre-cane task	Cane task	Post-cane task	ANOVA		
					Task	Group	Interaction
V-COP (mm/s)	LG	53.07 ± 8.99	42.79 ± 7.83^*^	46.17 ± 7.59^*^	*P* < 0.01	N.S.	*P* < 0.01
	DC	53.77 ± 9.22	25.23 ± 4.11^*^	55.02 ± 8.67^#^			
EMG-TA (% MVC)	LG	23.80 ± 3.70	17.79 ± 2.57^*^	22.40 ± 2.76	*P* < 0.01	N.S.	*P* < 0.01
	DC	23.83 ± 3.68	5.46 ± 1.27^*^	24.58 ± 3.22^#^			
EMG-GAS (% MVC)	LG	27.56 ± 2.87	24.72 ± 3.01	27.12 ± 3.77	*P* < 0.01	N.S.	N.S.
	DC	30.31 ± 4.63	18.74 ± 2.86^*^	33.21 ± 7.86^#^			
CCI (% MVC)	LG	34.50 ± 3.90	26.53 ± 2.96^*^	31.69 ± 3.60	*P* < 0.01	N.S.	*P* < 0.01
	DC	34.24 ± 4.00	7.36 ± 1.86^*^	33.62 ± 3.63^#^			

Values are expressed as mean ± standard error of the mean in each group

*P < 0.05 (versus pre-cane task); ^#^ *P* < 0.05 (versus cane task)

Regarding muscle activity (Table 1), although TA and GAS activities were significantly affected by the tasks, the interaction between the tasks and the groups exhibited statistical significance only in the TA activity. Post hoc test revealed that although the TA activity in the LG group significantly decreased during the cane task compared to the pre-cane task, the GAS activity was not significantly different between the tasks. The TA and GAS activities in the DC group were significantly decreased during the cane task compared to the pre- and post-cane tasks. For the CCI of the GAS-TA (Table 1), the factors of tasks and the interaction between the tasks and the groups exhibited statistically significant effects. Post hoc test revealed that CCI in the LG group significantly decreased during the cane task compared to the pre-cane task. Further, it did present large sized effect (*r* = 0.65). Although post hoc test did not reveal a significant effect in CCI between the pre- and post-cane tasks, it did present a medium effect (*r* = 0.39). CCI in the DC group was significantly decreased during the cane task compared to the pre- and post-cane tasks. Although a large effect was seen in the cane versus pre-cane tasks (*r* = 0.91), a small effect in the pre- versus post-cane tasks (*r* = 0.08) was seen in the DC group.

Although the relative change in V-COP during the cane and post-cane tasks versus the pre-cane task was not significant, there was a significant interaction between the tasks and the groups (Table 2). Therefore, although the decreased postural sway induced by the cane use increases immediately after its withdrawal regardless of the supporting condition (lightly gripping or strongly supporting one’s own weight), the increase in postural sway occurred greatly when the participants strongly support their own weight with the cane. The relative change in CCI during the cane and post-cane tasks compared with the pre-cane task showed a significant effect of the tasks, the groups, and the interaction between them (Table 2). Therefore, we determined the following: (1) the relative change in co-contraction of the ankle-joint muscles versus the pre-cane task differed between the cane use conditions (lightly gripping versus. strongly supporting one’s own weight); (2) decreased co-contraction induced by the cane use increased immediately after its withdrawal; and (3) this increase in co-contraction rate was greater when the cane was used to support one’s own weight.

**Table 2:** Relative change (%) of V-COP and CCI during cane and post-cane tasks compared with pre-cane task

Variables	Group	Pre-cane task versus		ANOVA		
		Cane task	Post-cane task	Task	Group	Interaction
V-COP	LG	−19.55 ± 4.14	−13.08 ± 3.57	*P* < 0.01	N.S.	*P* < 0.01
	DC	−50.13 ± 4.82	7.18 ± 9.29			
CCI	LG	−7.96 ± 3.07	−2.81 ± 2.19	*P* < 0.01	*P* = 0.03	*P* < 0.01
	DC	−26.88 ± 3.96	−0.63 ± 2.82			

Values are expressed as mean ± standard error of the mean in each group

**Fig. 3:**
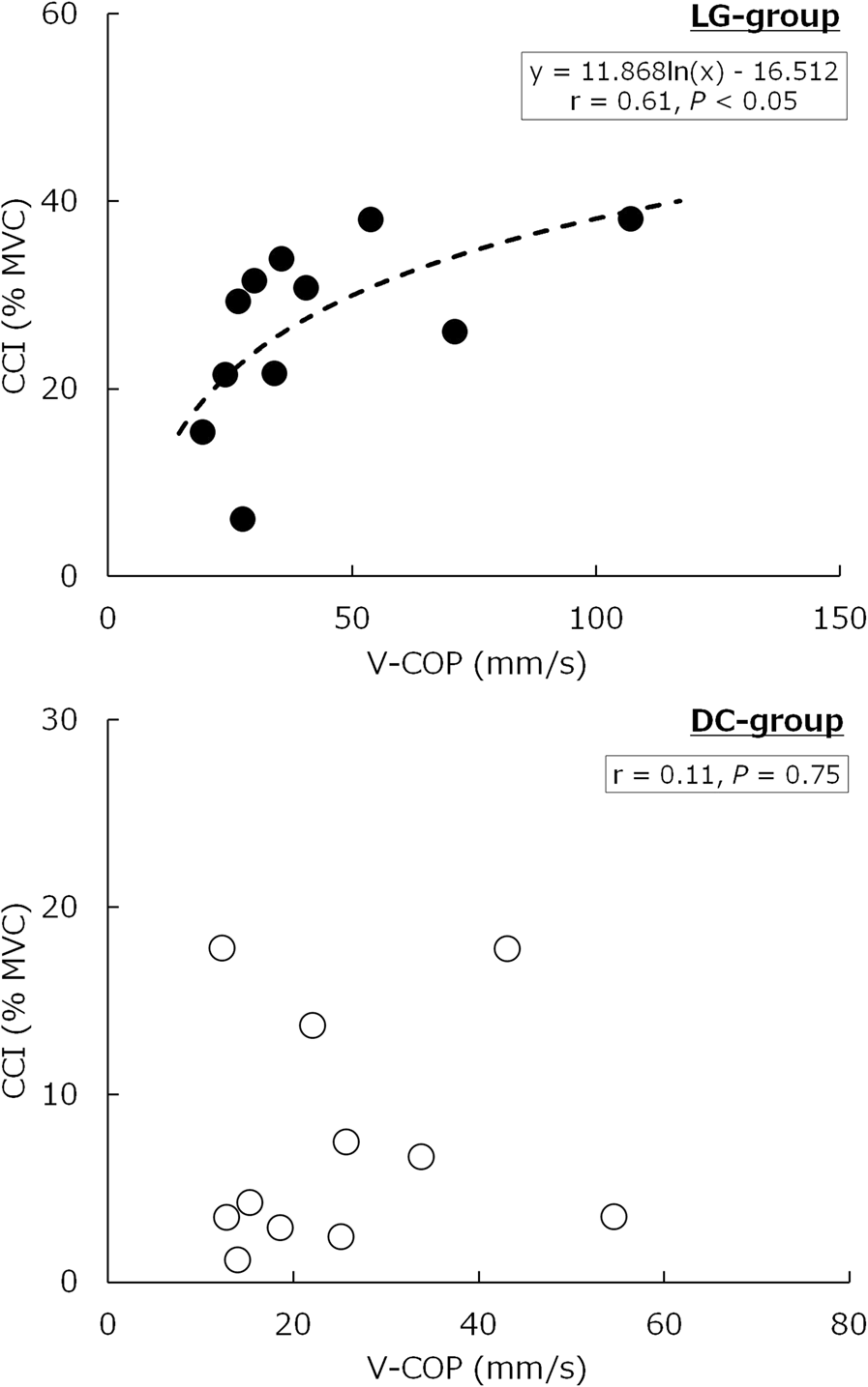
Relationship between the mean velocity of the center of foot pressure (V-COP) and the co-contraction index (CCI) of the ankle-joint muscles during the cane task

Regression curves between V-COP and CCI during the cane task were obtained from a nonlinear logarithmic regression analysis (Fig. 3). Although the equations of the regression curves in the LG group was *y* = 11.868 ln (*x*) − 16.512 (*P* < 0.05), no significant relationship was observed between V-COP and CCI in the DC group. Therefore, the relationship between postural sway and muscle activity varies according to the cane use condition.

## Discussion

The present study showed that postural sway was significantly decreased by the light gripping a cane. Contact of the finger or hand with an object has been shown to decrease postural sway during a quiet stance. The time of postural stabilization was measured after the participants made light finger contact with a fixed external object by Rabin et al. [4], who observed that fingertip contact forces stabilized with a time constant of <0.5 s, and a sway amplitude of COP stabilization rapidly occurred after fingertip contact. Further, the stereotypical pattern of force changes at the fingertip correlated with changes in COP by approximately 300 ms and was evident within the first 0.5 s of finger contact. On the other hand, Kouzaki and Masani [5] found that the effects of a light touch during a quiet stance were diminished due to loss of finger haptic feedback induced by tourniquet ischemia. Therefore, the light gripping a cane during SLS provides information about movement of the body segments by helping an individual senses movements of the trunk, arms, and thighs relative to one another in the LG group.

For the muscle activity, no significant effect of plantar flexor muscle (GAS) activity was observed in the LG group. Watanabe et al. [6] reported that plantar flexor muscle activity was not significantly changed by light touch on a wall during the Romberg stance (feet together). During SLS, no significant difference in plantar flexor muscle activity between with and without visual sensory input was observed [14]. However, dorsiflexor muscle (TA) activity during SLS was significantly lower with visual sensory input than without it. This study also observed that the TA activity in the LG group was lower in the cane task (i.e., with haptic sensory input through a cane) than in the pre-cane task. Therefore, the CCI of the ankle-joint muscles of the participants in the LG group was also lower in the cane task than in the pre-cane task. Increased co-contraction of the ankle-joint muscles during standing can increase postural stiffness. This postural stabilization strategy was observed under threatening conditions (platform height is low or high and toes are positioned at or away from the edge) in healthy normal individuals while standing [16, 17]. Haptic sensory input through a light touch provides information about the relative changes in one’s own body segments and allows one to stand easily compared with standing with no haptic sensory information. Regarding light touch effects on muscle activity between agonist and antagonist muscles, a previous study observed that antagonist muscle activity was decreased when utilizing light touching [9]. Another study suggested that this co-activation with light touch during standing might influence anticipatory postural adjustments (APAs) [18]. Therefore, these previous studies and the present study suggest that lightly gripping a cane decreases postural sway by decreasing co-contraction of the ankle-joint muscles.

An interesting finding of the present study is that, although postural sway decreased during the cane task, it also decreased immediately after cane withdrawal (in the post-cane task). However, this effect was not observed in the DC group. During the cane task, the participants in the LG group had to control their own bodies using their own muscle force since they were not allowed to apply force on the cane for support. On the other hand, the participants in the DC group were not required to control their own bodies using their own muscle force output; rather, they lightly gripped the cane. Therefore, although a significant relationship between postural sway and muscle activity (CCI) during the cane task was observed in the LG group, it was not observed in the DC group. The effect of motor learning is enhanced by providing additional feedback information, such as visual, auditory, haptic, and multimodal [19]. Further, the effectiveness of additional feedback information is also reported during postural control training [20]. The light gripping of a cane provides such feedback information because an individual can sense movements of the trunk, arms, and thighs relative to one another through a haptic sensory input. Although this study investigated just the immediate after-effect of lightly touching a cane, postural sway also decreased immediately after cane withdrawal. Therefore, these results suggest that the haptic sensory cue during postural maintenance might be promoted as a practice effect of postural control.

However, muscle activities (GAS, TA, and CCI) during the post-cane task were not significantly different from those of the pre-cane task in the LG group despite the fact that postural sway in the post-cane task remains lower than that of the pre-cane task. Therefore, other factor(s) such as the involvement of the nervous system or other muscles might play a role in the after-effect of cane withdrawal. A functional magnetic resonance imaging study reported that the neural activity during motor learning training is different between the kinds of feedback [21]. One prosthetics study suggested that haptic information is important in the acquisition of motor control, since the availability of haptic information through electrical stimulation can promote sensory-motor function recovery [22]. Further, they reported that the sensorimotor or prefrontal motor cortex activity during control of one’s own limbs differs between with and without haptic sensory feedback conditions. Therefore, further studies are needed to clarify the relationship between brain-nerve function or other muscle activities and postural sway during and after light touch.

The association between postural sway and light touch is scientifically interesting. However, it cannot be studied in various movements (i.e., daily activities) during contact with a fixed stable object. The present study revealed that postural sway was decreased not only by supporting one’s weight on a real cane but also by just lightly gripping it. A cane can be used while performing various movements. Therefore, our results suggest a potential new use for a cane. If individuals want to maintain their posture, they can support their body weights by strongly holding onto objects (e.g., a wall, handrail, or cane). This can help them stand independently while greatly reducing their risk of falling when faced with progressive leg weakness. However, this may cause weakened muscle function in persons who otherwise have sufficient muscle strength to stabilize their own postures because using a cane greatly reduces the muscular force outputs needed for posture stability as shown by this study. In contrast, lightly gripping a cane requires one to control one’s own body using muscle force while maintaining posture because contact force levels are insufficient for providing mechanical body support. Furthermore, the present study revealed that if a participant lightly gripped a cane, postural sway decreased not only during the light gripping but also immediately after the withdrawal of the cane. This result suggests that light touching is a potential training tool for acquiring postural control ability. If the effect of lightly gripping a cane on postural control is also relevant during various motions, lightly touching a cane might be useful for improving human movement.

## Conclusions

This study investigated the effect and after-effect of lightly gripping a cane on postural sway and muscle activity. Postural sway is decreased by light gripping and is accompanied by decreased co-contraction of the ankle-joint muscles, especially TA activity. Further, although postural sway also decreased immediately after the withdrawal of a lightly gripped cane, this effect was not observed when a person used a cane to support his weight. A lightly gripped cane provides a haptic sensory cue that can be used to assist postural control mechanisms due to enhanced perception of self-motion through sensory interaction with the environment through the cane. Furthermore, the haptic sensory cue during postural maintenance might be promoted as a practice effect of balance control ability. Therefore, our results suggest a potential new use for a cane.

## Abbreviations

ANOVA: analysis of variance
CCI: co-contraction index
COP: center of foot pressure
DC group: depend-on-cane group
EMG: electromyography
GAS: gastrocnemius
LG group: light-grip group
MVC: maximum voluntary contraction
RMS: root mean square
SLS: single-leg stance
TA: tibialis anterior
V-COP: mean velocity of COP trajectory
